# Integrating host gene expression and the microbiome to explore disease pathogenesis

**DOI:** 10.1186/s13059-015-0625-1

**Published:** 2015-04-08

**Authors:** Allyson L Byrd, Julia A Segre

**Affiliations:** Microbial Genomics Section, Translational and Functional Genomics Branch, National Human Genome Research Institute, Bethesda, MD 20892 USA

## Abstract

In a recent study, rich clinical assessment and longitudinal study design are combined with host gene expression and microbial sequencing analyses to develop a framework for exploring disease etiology and outcomes in the context of human inflammatory disease.

See related article: http://dx.doi.org/10.1186/s13059-015-0637-x

## Clinical studies exploring the role of the microbiome in disease outcome

Modulating the microbiome through prebiotics, probiotics and antibiotics holds tremendous potential for the treatment and prevention of human inflammatory disorders, but there is a need for medical research to explore how alterations in microbial communities are associated with disease state and host physiology. Few diseases have an easily explained etiology as outcomes are affected by a myriad of factors, including host genetics, microbial communities and environmental factors. Well-designed longitudinal human clinical studies are necessary to move from ‘correlation’ to ‘causation’. However, integrating rich clinical metadata with large datasets characterizing microbial and host attributes is a daunting task. A recent article by Morgan, Kabakchiev and colleagues lays out the analytic framework necessary to achieve this goal [1].

In their study, a complex set of interwoven diseases and outcomes is explored simultaneously to tease out the contributions to clinical outcome of several factors: underlying disease, host gene expression and mucosal microbiome composition [1]. Morgan *et al*. analyzed samples from ulcerative colitis (UC) and familial adenomatous polyposis (FAP) patients who had undergone ileal pouch-anal anastomosis (IPAA) surgery or so-called ‘J-pouch’ construction. Genetically, the auto-inflammatory nature of UC is associated with polymorphisms in 160 genes, whereas FAP displays a more classic Mendelian inheritance linked to mutations in a single gene [2]. Outcomes of IPAA surgery vary widely between these conditions - approximately half of UC patients experience an episode of pouchitis (inflammation of the ileal pouch), whereas this outcome is rare in FAP patients [3]. This clinical discrepancy suggests a potential role of host genetics in the onset of pouchitis. However, the gut microbiome also likely plays a role in disease pathogenesis as pouchitis has been successfully treated with antibiotics and might be prevented through the use of probiotics [3]. With known genetic and microbial influences, pouchitis is an ideal model for exploring the effects of host-microbe interactions on clinical outcome.

## Data dimension reduction to achieve maximum power from clinical studies

Previous studies have looked at microbial communities and host transcriptomes in patients who have undergone IPAA; however, thus far none has studied them simultaneously to explore host-microbe interactions. In their study, Morgan and colleagues measured host gene expression and microbial community composition in paired biopsies (pouch/afferent limb) from 265 patients undergoing IPAA because of UC or FAP [1]. They used microarrays and 16S ribosomal RNA gene sequencing to interrogate 19,908 host transcripts and 6,999 observed operational taxonomic units (OTUs; a proxy for bacterial species). Ribosomal sequences with >97% sequence identity were grouped together as an OTU.

Identifying associations between datasets of this size requires an unreasonably large sample size or the compression of many variables into a few. The required size of the population or the degree of compression can be calculated with power equations. In their study, Morgan *et al*. elegantly explain how, given their sample size and the desire to retain 90% power and an alpha equal to 0.05, thousands of variables must be reduced to approximately 100 transcripts and 100 bacterial clades. From these 10^4^ pairwise tests, statistically significant conclusions between the datasets could be drawn.

To achieve the data compression required by the power equation, Morgan and colleagues employed various dimension-reduction techniques [1]. The issue of data reduction is not new to the genomic community, especially as large datasets become the norm. Reduction analysis methods are common in expression quantitative trait loci (eQTL) and microarray studies [4,5]. By combining multiple SNPs into haplotypes, the HapMap project was able to reduce the overall number of tags and increase the power of their analyses [6].

The microbial OTUs were first filtered for abundance and then subjected to unsupervised and supervised cluster analyses. For unsupervised dimensionality reduction, OTUs were transformed into nine clade principal components (cPCs), which explained 50% of the observed variance [7]. For supervised reduction, filtered OTUs were hierarchically clustered, and the OTU with the lowest mean abundance was chosen to represent the cluster. Transcripts were also subjected to supervised and unsupervised dimension reduction. In the supervised approach, transcripts were chosen based on their previous implications in inflammatory bowel disease (IBD), pouchitis or host-microbe interactions and were then clustered based on expression profiles to yield 75 gene medoids - gene(s) with similar expression patterns. For unsupervised reduction, transcripts with stable expression across all subjects were discarded, and the remaining transcripts were compressed into nine gene principle components (gPCs). In the end, thousands of host transcripts and OTUs were reduced down to 138 features - a number well within the limit imposed by the power equation.

## Drawing meaningful connections between host expression, microbial composition and clinical metadata

In addition to transcriptome and microbiome data, extensive metadata, including antibiotic usage, score of inflammation and post-surgical outcomes, were collected for each patient [1]. To uncover connections among these different types of data, Morgan and colleagues used various linear modeling techniques. With these methods, they validated previous findings that host transcript variation is best explained by biopsy location [8], and microbial community variation is best explained by an individual's antibiotic usage. When they controlled for antibiotic usage, very few microbial taxa could be associated with inflammation or clinical outcome, with the exception of *Escherichia* and *Actinobacteria*, which are positively and negatively associated with inflammation, respectively.

The novelty of this research is the incredibly rich, well-performed clinical study and the depth of analyses focused on host-microbe interactions. Such comparisons would not have been possible without the supervised and unsupervised data reduction methods. Using multivariant linear modeling to control for antibiotics, inflammation and outcome, Morgan and colleagues measured gene-clade associations [1]. Surprisingly, transcript-microbe interactions were modest - only two gPCs associated with four cPCs. Loadings of the gPCs corresponded to proteins in the complement cascade and interleukin-12 pathway, whereas several clades that featured in the four cPCs could be linked back to antibiotic usage, including increased Enterobacteriaceae and decreased *Bacteroides* and Firmicutes. Overall, this modest number of interactions indicates that microbial composition is more likely shaped by early-life colonization or diet than by local gene expression [9].

Next, Morgan and colleagues applied the gene-medoids and representative clades from supervised clustering to linear discriminant analysis (LDA) [1]. They wanted to evaluate whether various combinations of genes and microbes could be used as markers to discriminate between different clinical outcomes. After restricting the analysis to samples without antibiotic usage, LDA analysis was able to discriminate between FAP patients and those with Crohn’s disease-like inflammation but not between other outcomes, such as acute or no pouchitis.

The dominant effect of antibiotic usage was a common theme throughout this paper. Antibiotics were the main driver of microbial community composition and highly predictive of the chronic pouchitis outcome, which is unsurprising given that antibiotics are often prescribed to treat pouchitis [3]. Antibiotics altered microbial compositions so strongly that the effects of other factors such as inflammation, host genetics and clinical outcome were often overshadowed, and LDA was unable to identify any microbes or genes predictive of outcome.

## Utilizing clinical data to generate testable hypotheses

A common goal of clinical studies is to better stratify and predict disease outcomes. In this particular study, that goal was not realized owing to complications imposed by the patients’ antibiotic usage history and lack of multiple time-points - factors that limit many clinical studies. For human treatment studies, many factors confounding analysis cannot be controlled without placing an undue burden on the patient - that is, asking patients to provide frequent biopsies or to refrain from taking antibiotics. Thus, when available, animal models of a disease can be useful for well-controlled explorations of hypotheses generated from clinical studies. A recent paper by Buffie and colleagues elegantly demonstrated how hypotheses from *Clostridium difficile* colonization-resistance studies in humans could be investigated with a mouse model [10]. This human-mouse study integration led to the discovery of single bacterial specie(s) that confer colonization resistance to the pathogen *C. difficile*. Ideally, these results will soon be brought back to humans and tested in a controlled clinical setting. Clinical studies are essential to generate hypotheses about what should be modeled and tested in animals and then to validate concepts derived from mouse studies in an iterative fashion.

## Concluding remarks: advantages and pitfalls of growing data sets

Morgan and colleagues used 16S amplicon sequencing and microarrays to infer host-microbe interactions [1]. In the future, microbial communities will be described with whole-genome shotgun sequencing metagenomics, and transcriptomes will be captured with deeper RNA-Seq. These new technologies will generate data at a greater resolution than ever before, while simultaneously generating new analysis conundrums. Microbial and clinical experts will need to combine forces with computational biologists and statisticians to develop methods for analyzing these very large datasets in order to be able to visualize both the ‘forest’ and the ‘trees’. When designing clinical studies characterized by large-scale sequencing technologies, researchers will need to maximize the large cohorts necessary for discovery-driven research and smaller cohorts sufficient to explore defined clinical questions.

## Abbreviations

cPC: Clade principal component
eQTL: Expression quantitative trait loci
FAP: Familial adenomatous polyposis
gPC: Gene principle component
IBD: Inflammatory bowel disease
IPAA: Ileal pouch-anal anastomosis
LDA: Linear discriminant analysis
OTU: Operational taxonomic unit
UC: Ulcerative colitis
